# Responses in colonic microbial community and gene expression of pigs to a long-term high resistant starch diet

**DOI:** 10.3389/fmicb.2015.00877

**Published:** 2015-08-25

**Authors:** Yue Sun, Liping Zhou, Lingdong Fang, Yong Su, Weiyun Zhu

**Affiliations:** Jiangsu Key Laboratory of Gastrointestinal Nutrition and Animal Health, College of Animal Science and Technology, Nanjing Agricultural UniversityNanjing, China

**Keywords:** raw potato starch, pig, transcriptional profiling, colon, mucosa-associated microbiota

## Abstract

Intake of raw potato starch (RPS) has been associated with various intestinal health benefits, but knowledge of its mechanism in a long-term is limited. The aim of this study was to investigate the effects of long-term intake of RPS on microbial composition, genes expression profiles in the colon of pigs. Thirty-six Duroc × Landrace × Large White growing barrows were randomly allocated to corn starch (CS) and RPS groups with a randomized block design. Each group consisted of six replicates (pens), with three pigs per pen. Pigs in the CS group were offered a corn/soybean-based diet, while pigs in the RPS group were put on a diet in which 230 g/kg (growing period) or 280 g/kg (finishing period) purified CS was replaced with purified RPS during a 100-day trial. Real-time PCR assay showed that RPS significantly decreased the number of total bacteria in the colonic digesta. MiSeq sequencing of the V3-V4 region of the 16S rRNA genes showed that RPS significantly decreased the relative abundance of *Clostridium, Treponema, Oscillospira, Phascolarctobacterium*, RC9 gut group, and S24-7-related operational taxonomic units (OTUs), and increased the relative abundance of *Turicibacter, Blautia, Ruminococcus, Coprococcus, Marvinbryantia*, and *Ruminococcus bromii*-related OTUs in colonic digesta and mucosa. Analysis of the colonic transcriptome profiles revealed that the RPS diet changed the colonic expression profile of the host genes mainly involved in immune response pathways. RPS significantly increased proinflammartory cytokine IL-1β gene expression and suppressed genes involved in lysosome. Our findings suggest that long-term intake of high resistant starch (RS) diet may result in both positive and negative roles in gut health.

## Introduction

It has been known that starches are one of the major carbohydrates available in the human colon (Anderson et al., 1981). Starch that escapes digestion in the small intestine (human and animal) can enter the large intestine, where it is used as a substrate for bacterial fermentation (Topping and Clifton, 2001). The effects and potential health benefits of resistant starch (RS) have been extensively studied in recent years (Martinez-Puig et al., 2003; Fung et al., 2012). Bacterial fermentation of RS results in the production of short-chain fatty acids (SCFAs), mainly acetate, propionate and butyrate, which are considered to have beneficial effects upon hindgut health (Young et al., 2005; Le Leu et al., 2009). In addition, RS is considered to have a role in regulating the adipose metabolism of humans and animals, and has positive effects on the health of the body (So et al., 2007). The addition of raw potato starch (RPS) to the diet of pigs could increase the amount of starch entering the large bowel because the native granular structure of this starch, which became an available source for hindgut microflora (Martinez-Puig et al., 2003; Fang et al., 2014).

A study by Haenen et al. (2013b) showed that diets high in RS affected the microbiota composition in the colon of pigs, where butyrate-producing bacteria including *Faecalibacterium prausnitzii* and *Ruminococcus bromii* were stimulated in abundances. *In vitro* studies also found that populations related to *R. bromii* were the primary starch degrader, while bacteria related to *Prevotella* spp., *Bifidobacterium adolescentis*, and *Eubacterium rectale* might be further involved in the trophic chain (Kovatcheva-Datchary et al., 2009). In a recent study, *R. bromii* was confirmed to possess an exceptional ability to colonize and degrade starch particles when compared with previously studies (Ze et al., 2012). While most of the study focused on the microbiota in the gut digesta, mucosa-associated microbiota were believed to be more closely related to the gut function of the host. Despite the importance of the mucosal community for bacterial intestinal colonization, pathogen resistance, and host-microbiota cross-talk (Leser and Mølbak, 2009), the porcine mucosa-associated microbiota and their diet-related changes were not well investigated at this degree until now.

Recently, genome-wide transcriptional profiling is extensively used to investigate how animals or humans respond to their diets, which contributes to our understanding of the mechanism of a healthy diet. Microarray assay showed that long-term ingestion of a rapidly digestible starch (high amylopectin ratio) significantly elevated hepatic lipogenesis, which is associated with a higher serum insulin concentration and more lipogenic genes being expressed in the liver (Jun et al., 2010). It has been recently reported that consumption of a diet high in RS for 2 weeks induced catabolic pathway but suppressed immune and cell division pathways in the proximal colon of male pigs (Haenen et al., 2013a). Information on the long-term effects of RS on the gene expression profiles of pigs, however, remains limited.

In this study, we postulated that a long-term intake diet high in RS could change the microbial composition in both colonic digesta and mucosa, modulate mucosal transcriptomes, and eventually influence host health. Because of its similar homology to human, the pig has been recognized as one of the ideal models for the study of human nutrition (Guilloteau et al., 2010). Thus, by using RPS to substitute about half of the corn starch (CS) in the diet, the present study aimed to investigate the effects of long-term intake of RPS on digesta- and mucosa-associated microbial composition and gene expression profiles in the colon of pigs.

## Materials and methods

### Animals, housing, and diets

This study was approved by the Nanjing Agricultural University Animal Care and Use Committee. The treatment, housing, husbandry and slaughtering conditions conformed to the Experimental Animal Care and Use guidelines of China (Chinese Science and Technology Committee, 1988). All pigs were raised on a commercial farm in the Jiangsu Province of China. Thirty-six Duroc × Landrace × Large White growing barrows (70 days of age, 23.78 ± 1.87 kg) were randomly allocated to two groups, each group consisting of three pigs per pen, and six replicates. Pigs in the control group were offered a corn/soybean-based diet, while 230 g/kg purified CS was replaced with purified RPS in the RPS diet group. Diets were formulated (Table 1) according to the nutrient requirements of the National Research Council (1998). When animals reached the age of 120 days, diets were adapted to the nutrient requirements of the animals (finishing diet) and the amount of purified starch increased to 280 g of CS or RPS per kilogram of feed. Pigs had unlimited access to feed and water throughout the experimental period, which consisted of two 50-day trials in which the pigs consumed the growing diet (days 0–50) and finishing diet (days 51–100), respectively.

**Table 1:** **Composition and nutrient analysis of experimental diets (as-fed basis)**.

**Diets**	**Growing pigs**		**Finishing pigs**	
	**CS**	**RPS**	**CS**	**RPS**
**INGREDIENTS (g/kg)**				
Corn starch	230.0	–	280.0	–
Raw potato starch	–	230.0	–	280.0
Corn	360.0	360.0	360.0	360.0
Wheat bran	90.0	90.0	120.0	120.0
Soybean meal	250.0	250.0	210.0	210.0
Extruded soybean	30.0	30.0	–	–
Soybean oil	8.00	8.00	–	–
Dicalcium phosphate	9.80	9.80	8.80	8.80
Limestone	7.80	7.80	7.70	7.70
Salt	3.00	3.00	3.00	3.00
Vitamin and mineral premix^a^	10.0	10.0	10.0	10.0
L-Lysine	1.00	1.00	0.50	0.50
L-Methionine	0.40	0.40	–	–
**NUTRIENT ANALYSIS (g/kg)**				
CP	174.5	174.5	147.3	147.3
Starch	505.6	504.5	550.2	549.5
Resistant starch	6.40	133.5	5.20	153.5
Ash	72.1	73.2	61.0	61.6
NDF	95.77	95.78	102.5	102.6

a *This mineral and vitamin premix (1%) supplies per kg diet as follows: VA 11 000 IU, VD3 1 000 IU, VE 16 IU, VK1 1 mg, VB1 0.6 mg, VB2 0.6 mg, d-pantothenic acid 6 mg, nicotinic acid 10 mg, VB12 0.03 mg, folic acid 0.8 mg, VB6 1.5 mg, choline 800 mg, Fe 165 mg, Zn 165 mg, Cu 16.5 mg, Mn 30 mg, Co 0.15 mg, I 0.25 mg, Se 0.25 mg*.

### Sampling

On day 100, one pig from each replicate was slaughtered when it approached target slaughter weight (105–110 kg). Feed was withheld from the pigs 12 h before slaughter. Pigs were weighed and transported (10 km) to a local commercial slaughterhouse, and slaughtered via electrical stunning followed by exsanguination. The animals were bled and opened immediately, and the proximal colon was excised for the collection of digesta and mucosa samples. The pH value of digesta was determined using a handheld pH meter (HI 9024C; HANNA Instruments, Woonsocket, RI). The colon tissues were washed with sterile phosphate-buffered saline (PBS) (pH 7.0). Mucosal samples were collected by scraping the luminal surface with a sterile glass slide. All samples were kept in liquid nitrogen for further gene expression and microbial community analysis.

### DNA extraction, PCR amplification and illumina MiSeq sequencing

The total genomic DNA was isolated from colonic digesta and mucosa using a commercially available stool DNA extraction kit according to the manufacturer's instructions of (QIAamp DNA Stool Mini Kit, Qiagen, Hilden, Germany). The concentration of extracted DNA was determined by using a Nano-Drop 1000 spectrophotometer (Thermo Scientific Inc., Wilmington, DE, USA). The V4-V5 region of the bacterial 16S ribosomal RNA gene was amplified by polymerase chain reaction (PCR) using bacterial universal primers 515F and 907R with an eight-base sequence unique to each sample as a barcode (Lane et al., 1985; Kroes et al., 1999). The amplification program consisted of an initial denaturation step at 95°C for 2 min, followed by 25 cycles at 95°C for 30 s, 55°C for 30 s, and 72°C for 30 s and a final extension at 72°C for 5 min. PCR reactions were performed in triplicate 20 μl mixture containing 4 μl of 5 × FastPfu Buffer, 2 μl of 2.5 mM dNTPs, 0.8 μl of each primer (5 μM), 0.4 μl of FastPfu Polymerase, and 10 ng of template DNA.

Because one mucosal DNA sample from RPS group failed to be amplified by PCR, amplicons from other 23 samples were extracted from 2% agarose gels and purified using the AxyPrep DNA Gel Extraction Kit (Axygen Biosciences, Union City, CA, USA) according to the manufacturer's instructions and quantified using QuantiFluor™-ST (Promega, USA). Purified amplicons were pooled in equimolar and paired-end sequenced (2 × 250) on an Illumina MiSeq platform according to the standard protocols.

### Bioinformatics analysis

Raw fastq files were demultiplexed and quality-filtered using QIIME (version 1.17) with the following criteria: the 250 bp reads were truncated at any site receiving an average quality score < 20 over a 10 bp sliding window, discarding the truncated reads that were shorter than 50 bp; exact barcode matching, 2 nucleotide mismatch in primer matching, reads containing ambiguous characters were removed; and only sequences that overlap longer than 10 bp were assembled according to their overlap sequence. Reads that could not be assembled were discarded.

Operational taxonomic units (OTUs) were clustered with 97% similarity cutoff using UPARSE (version 7.1 http://drive5.com/uparse/) and chimeric sequences were identified and removed using UCHIME. To assess bacterial diversity among samples in a comparable manner, a randomly selected, 43274-sequence (the lowest number of sequences in the 23 samples) subset from each sample was compared for the phylogenetic affiliation by RDP Classifier (http://rdp.cme.msu.edu/) against the Silva (SSU115) 16S rRNA database using a confidence threshold of 70% (Amato et al., 2013). We also calculated the coverage percentage using Good's method (Good, 1953), the abundance-based coverage estimator (ACE), the bias-corrected Chao richness estimator, and the Shannon and Simpson diversity indices using the MOTHUR program (http://www.mothur.org) (Schloss et al., 2009). The raw pyrosequencing reads were submitted to Sequencing Read Archive (SRA) database under the accession id: SRA061866. Genera (OTUs) with relative abundances higher than 0.05% within total bacteria were defined as predominant genera (OTUs), and sorted for comparing the difference among different groups.

### Real-time PCR

Primer set Bact1369/Prok1492 was used for the quantification of total bacteria by real-time PCR on an Applied Biosystems 7300 Real-Time PCR System (Applied Biosystems, USA) using SybrGreen as the fluorescent dye (Suzuki et al., 2000). A reaction mixture (25 μl) consisted of 12.5 μl of IQ SYBR Green Supermix (Bio-Rad), 0.2 μM of each primer set and 5 μl of the template DNA. The amount of DNA in each sample was determined in triplicate, and the mean values were calculated.

### RNA extraction and purification

The total RNA of colonic mucosa was extracted using Trizol reagent (Life Technologies, Carlsbad, CA, USA) following the manufacturer's instructions, and were checked for RNA integrity and purity by an Agilent Bioanalyzer 2100 (Agilent Technologies, Santa Clara, CA, USA). The qualified total RNA was further purified by an RNeasy mini kit (Qiagen, Gmbh, Hilden, Germany) and an RNase-Free DNase Set (Qiagen, Gmbh, Hilden, Germany). Only those samples that had an OD260/OD280 ratio of approximately 2.0 and showed no degradation (RNA integrity number ≥ 7.0) were used to generate labeled targets.

### Microarray hybridization and data analysis

The RNA sample from each group was hybridized to an Agilent Porcine 4 × 44 K one-color gene expression microarray (catalog number 026440; Agilent Technologies, Santa Clara, CA, US) containing 43,603 probe sets, as described in the Gene Expression Omnibus (http://www.ncbi.nlm.nih.gov/geo/). Because there were six replicates from each group, four biological replicates were randomly selected for the microarrays to reduce the costs of the experiment. Total RNA was amplified and labeled using a Low Input Quick Amp Labeling Kit (Agilent Technologies, Santa Clara, CA, USA), following the manufacturer's instructions. Each slide was hybridized with 1.65 μg Cy3-labeled cRNA using a Gene Expression Hybridization Kit (Agilent Technologies, Santa Clara, CA, USA) in hybridization oven. After 17 h of hybridization, slides were washed with a Gene Expression Wash Buffer Kit (Agilent technologies, Santa Clara, CA, USA), following the manufacturer's instructions. The slides were then scanned on an Agilent Microarray Scanner (Agilent Technologies, Santa Clara, CA, USA), and data were extracted with Feature Extraction Software 10.7 (Agilent Technologies, Santa Clara, CA, USA). Raw data were normalized using a quantile algorithm, Gene Spring Software 11.0 (Agilent Technologies, Santa Clara, CA, USA). Array data have been submitted to the Gene Expression Omnibus under accession number GSE71305. Microarrays were performed by Shanghai Biochip Co., Ltd (Shanghai, China).

### Pathway and network analysis

The SBC Analysis System (SAS, http://sas.ebioservice.com) was used to further identify the differentially expressed genes between the two dietary groups, and genes with values of *P* < 0.05 were extracted. To further clarify the function of the differentially expressed genes in this study, transcripts were first annotated to pig (ssc) genes, and then other significant transcripts were annotated against human (hsa), mouse (mmu) and rat (rno) genes. Highly expressed genes in pigs fed the RPS diet that showed at least a 1.5-fold higher or lower expression level than in pigs fed the CS diet were selected for further study. In addition, the differentially expressed genes identified between the two groups were mapped to Gene Ontology (GO, http://www.geneontology.org/) terms and the Kyoto Encyclopedia of Genes and Genomes (KEGG, http://www.genome.jp/kegg/) pathways to identify potential pathways associated with dietary treatment. *P*-values < 0.05 and a false discovery rate < 0.05 were considered to be statistically significant. The gene network analysis of the differentially expressed genes involved in significant pathways was carried out using the KEGG database.

### Statistical analysis

Data were analyzed by SPSS 17.0 as a randomized block design, considering the diet as the main effect and the replicate as a block. The effects of diet and niche compartments on the colonic microbial community were tested for significance using a Two-Way ANOVA program. The effects of diet on gene expression in the colon were tested for significance using the Student's *t*-test. *P* values were corrected for multiple testing by using a false-discovery rate (*Q*-value) method (Benjamini and Hochberg, 1995). Significant differences were declared when *P* < 0.05.

## Results

### Microbiota analysis

**Figure 1:**
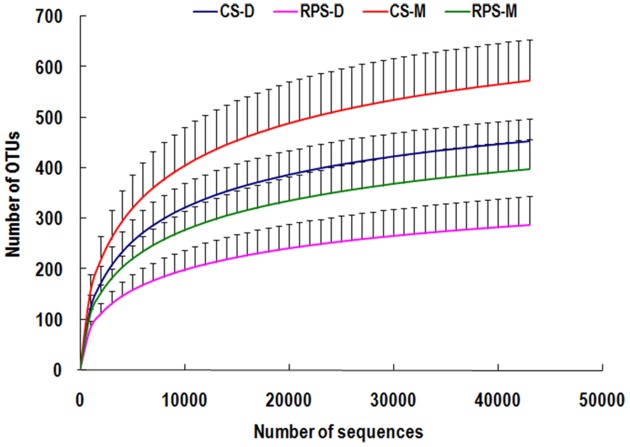
**Rarefaction curves comparing the number of sequences with the number of phylotypes found in the 16S rRNA gene libraries from microbiota in the colonic digesta (D) and mucosa (M) of pigs fed corn starch (CS) and raw potato starch (RPS) diets**.

Across all 23 samples, 1,397,445 quality sequences were classified as being bacteria with a read length higher than 250 bp. The average length of the quality sequences was 396 bp. The statistical estimates of species richness for 43,274-sequence subsets from each sample at a genetic distance of 3% are presented in Table 2. The richness estimators (ACE and Chao) of colonic microbiota were significantly affected by niche compartment and dietary treatment, while the diversity indices (Shannon and Simpson) were not affected by dietary treatment. The rarefaction curves generated by MOTHUR plotting the number of reads by the number of OTUs tended to approach the saturation plateau (Figure 1). The curves also showed that ACE and Chao indices from colonic mucosa were significantly higher than those from colonic digesta, and the indices from the CS diet group were significantly higher than those from the RPS group (*P* < 0.05).

**Table 2:** **Diversity estimation of the 16S rRNA gene libraries from microbiota in the colon of pigs fed corn starch (CS) and raw potato starch (RPS) diets**.

**Item**	**Colonic digesta**		**Colonic mucosa**		**SEM^a^**	**Effects (*****P*****-value)**		
	**CS**	**RPS**	**CS**	**RPS**		**Niche compartment**	**Dietary**	**Interaction**
Ace	581.8	381.3	685.8	494.0	133.2	0.001	< 0.001	0.884
Chao	589.3	383.2	699.6	498.7	137.6	0.001	< 0.001	0.929
Shannon	3.327	3.156	3.657	3.442	0.445	0.105	0.300	0.902
Simpson	0.102	0.087	0.091	0.086	0.042	0.721	0.616	0.929

a *SEM, standard error of means, n = 5 or 6 (one mucosal sample in RPS group was missing because it failed to be amplified by PCR)*.

**Figure 2:**
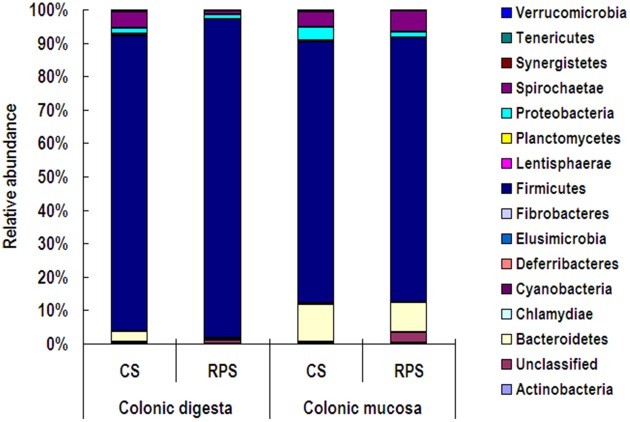
**Relative abundances of microbial phyla in the colonic digesta and mucosa of pigs fed corn starch (CS) and raw potato starch (RPS) diets**.

At the phylum level, Firmicutes was the predominant phylum with the abundance higher than 73% in both colonic digesta and mucosa, followed by the phyla Bacteroidetes and Spirochaetae (Figure 2). Compared to the colonic digesta, the relative abundances of Bacteroidetes and Synergistetes from colonic mucosa increased significantly, while the abundance of Firmicutes decreased significantly (*P* < 0.05). However, dietary treatment did not have an effect on the relative abundance of any phylum.

Genus-level analysis revealed that *Streptococcus*, uncultured Ruminococcaceae and *Lactobacillus* were the three most predominant genera in the colonic digesta and mucosa of pigs. As shown in Table 3, compared with colonic digesta, genera *Oscillospira, Parabacteroides, Phascolarctobacterium*, S24-7, *Alloprevotalla, Anaerotruncus*, RF16, *Bacteroides, Spirochaeta, Mucispirillum, Oscillibacter*, dgA-11 gut group, *Sutterella*, and *Mogibacterium* increased in relative abundance from colonic mucosa, whereas the abundances of *Turicibacter, Anaerotruncu*, and Peptostreptococcaceae incertae_sedis decreased (*P* < 0.05). The consumption of RPS significantly increased the abundances of *Turicibacter, Ruminococcus, Blautia, Coprococcus, Marvinbryantia*, and Lachnospiraceae incertae sedis, and decreased the abundances of *Clostridium*, S24-7, RC9 gut group, *Parabacteroides, Phascolarctobacterium, Oscillospira, Oscillibacter*, and *Mogibacterium* (*P* < 0.05).

**Table 3:** **Relative abundances of microbial genera (percentage) that were significantly affected by the dietary treatment or the niche compartment in the colon of pigs^a^**.

**Genus**	**Colonic digesta**		**Colonic mucosa**		**SEM^b^**	*****P***-value**		**Q-value**	
	**CS**	**RPS**	**CS**	**RPS**		**Niche compartment**	**dietary**	**Niche compartment**	**dietary**
*Alloprevotella*	0.167	0.064	0.566	0.857	0.456	0.001	0.579	0.007	0.693
*Anaerotruncus*	0.156	0.110	0.368	0.230	0.139	0.001	0.049	0.007	0.105
*Bacteroides*	0.072	0.024	1.555	0.160	0.824	0.004	0.016	0.021	0.051
*Blautia*	0.553	4.180	0.268	3.889	2.674	0.736	< 0.001	0.853	0.004
*Clostridium*	15.01	6.935	12.17	6.453	6.732	0.498	0.011	0.662	0.038
*Coprococcus*	0.018	0.205	0.044	0.404	0.187	0.046	< 0.001	0.116	0.002
Uncultured Coriobacteriaceae	0.118	0.059	0.137	0.053	0.084	0.828	0.045	0.896	0.010
Unclassified Family XIII	0.026	0.014	0.032	0.014	0.023	0.738	0.124	0.641	0.029
Lachnospiraceae incertae sedis	0.162	0.828	0.123	0.510	0.435	0.240	0.001	0.440	0.008
Unclassified Lachnospiraceae	1.235	3.654	0.876	3.337	2.795	0.760	0.037	0.853	0.092
*Marvinbryantia*	0.187	0.410	0.185	0.412	0.204	0.997	0.006	0.997	0.029
*Mogibacterium*	0.028	0.008	0.084	0.004	0.036	0.009	< 0.001	0.031	0.002
*Mucispirillum*	0.003	0.000	0.126	0.105	0.109	0.010	0.779	0.032	0.867
*Oscillibacter*	0.034	0.002	0.164	0.031	0.085	0.006	0.007	0.023	0.029
*Oscillospira*	0.093	0.022	0.590	0.250	0.263	< 0.001	0.006	0.001	0.029
*Parabacteroides*	0.220	0.044	1.115	0.290	0.482	< 0.001	0.001	0.002	0.005
Peptostreptococcaceae incertae sedis	9.662	13.314	5.432	6.857	4.794	0.004	0.125	0.021	0.213
*Phascolarctobacterium*	0.094	0.030	0.741	0.140	0.323	< 0.001	0.001	0.002	0.006
*Prevotella*	0.129	0.104	0.654	2.807	1.767	0.028	0.140	0.080	0.213
Prevotellaceae unclassified	0.118	0.005	0.629	0.100	0.284	< 0.001	< 0.001	0.005	0.004
Prevotellaceae uncultured	0.317	0.096	1.879	3.164	2.141	0.009	0.533	0.030	0.666
RC9 gut group	1.045	0.069	1.841	0.393	0.912	0.040	< 0.001	0.106	0.002
RF16	0.006	0.001	0.202	0.046	0.107	0.001	0.031	0.009	0.084
RF9	0.683	0.428	0.324	0.154	0.369	0.032	0.136	0.087	0.213
Unclassified Ruminococcaceae	0.748	0.382	0.865	0.679	0.316	0.090	0.023	0.216	0.071
*Ruminococcus*	0.594	3.120	0.856	4.537	2.900	0.452	0.009	0.654	0.032
S24-7 no rank	0.979	0.275	2.215	0.793	0.871	< 0.001	< 0.001	0.006	0.002
*Spirochaeta*	0.069	0.005	0.620	0.152	0.350	0.006	0.039	0.023	0.092
*Subdoligranulum*	0.141	3.621	0.238	1.900	2.947	0.506	0.032	0.662	0.084
*Sutterella*	0.000	0.000	0.097	0.018	0.057	0.006	0.061	0.023	0.120
*Turicibacter*	4.068	9.235	2.375	3.475	3.584	0.004	0.009	0.021	0.032
dgA-11 gut group	0.005	0.000	0.176	0.025	0.102	0.008	0.043	0.030	0.099
p-1088-a5 gut group	0.533	0.036	0.208	0.077	0.359	0.284	0.028	0.459	0.082

a *Genera with relative abundances higher than 0.05% within total bacteria were sorted and showed in the table*.

b *SEM, standard error of means, n = 5 or 6*.

At the OTU level, consumption of RPS diet significantly decreased the relative abundance of *Clostridium, Treponema, Oscillospira, Phascolarctobacterium*, RC9 gut group, and S24-7-related OTUs, and increased the relative abundance of *Turicibacter*, Lachnospiraceae, *Blautia, Ruminococcus, Coprococcus, Marvinbryantia*, and *Ruminococcus bromii*-related OTUs (Supplementary Table 1). Compared with the colonic digesta, *Phascolarctobacterium*, Bacteroidales, *Parabacteroides, Oscillospira*, Prevotellaceae, *Bacteroides*, and *Spirochaeta*-related OTUs increased significantly in the relative abundance in the colonic mucosa of pigs (*P* < 0.05).

**Figure 3:**
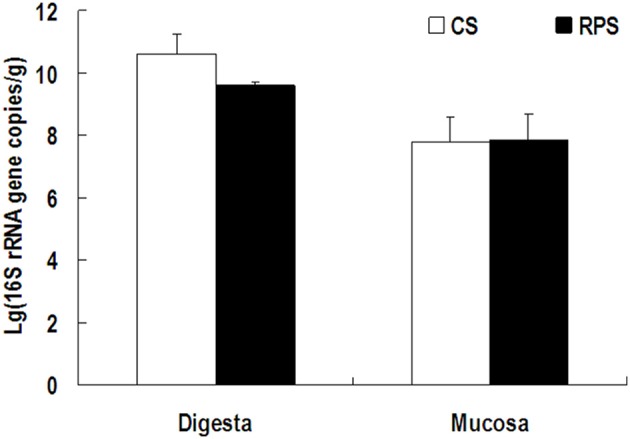
**The number of 16S rRNA gene copies of total bacteria in the colonic digesta and mucosa of pigs fed corn starch (CS) and raw potato starch (RPS) diets**.

**Figure 4:**
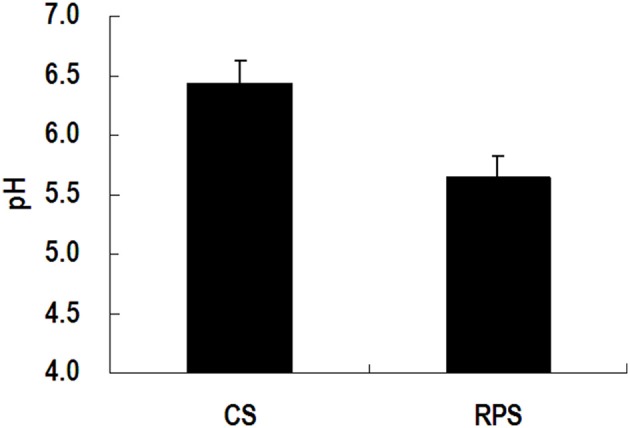
**The pH value in the colon of pigs fed corn starch (CS) and raw potato starch (RPS) diets**.

Because MiSeq sequencing analysis can only reflect the relative abundance of bacteria, quantitative real-time PCR was used to determine the completed 16S rRNA gene copies of bacteria in the colon of pigs (Figure 3). The consumption of RPS significantly decreased the total number of bacteria in the colonic digesta of pigs (*P* < 0.05), while there was no difference in colonic mucosa between the two dietary groups. In addition, the pH in the colon of pigs fed RPS diet was significantly lower than that in pigs fed CS diet (*P* < 0.05) (Figure 4).

### Transcriptome analysis

Microarrays were used to identify genes that were differentially expressed due to different dietary starch treatments. The analysis of gene expression data from colonic mucosal samples identified 781 differentially expressed genes with the fold change higher than 1.5 at a nominal *P*-value of 0.05. Of these 781 genes, 465 genes were up-regulated by the RPS diet, including 311 genes with functional annotation and 154 genes without functional annotation, whereas 316 genes were down-regulated, including 238 genes with functional annotation and 78 genes without functional annotation. The most induced gene was coagulation factor VII (F7), showing a 4.05-fold increase with the RPS treatment, whereas the most suppressed gene was dual oxidase 1 (DUOX1), with a 4.17-fold decrease (Supplementary Table 2).

**Figure 5:**
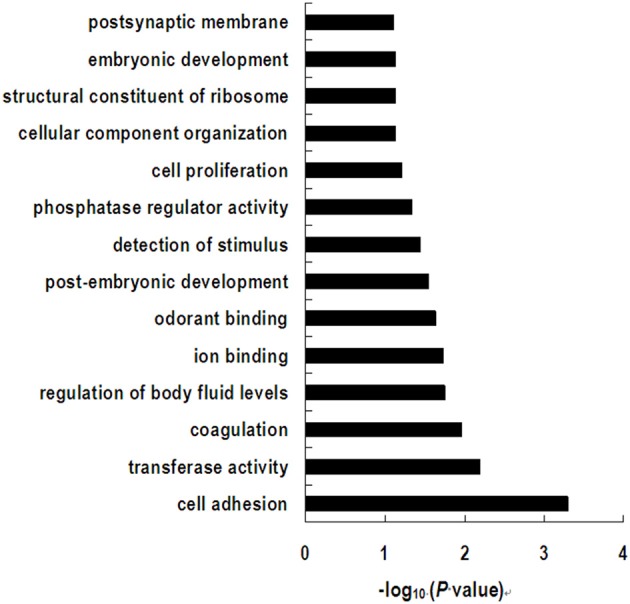
**Significant gene ontology terms represented on the list of differential expressed genes induced by dietary treatment**.

The differently expressed genes with functional annotation were included in a follow-up gene ontology (GO) and pathway analysis. GO category analysis showed that these genes were involved in a wide variety of physiological and biological events, such as cell adhesion, transferase activity, coagulation, regulation of body fluid levels, ion binding, and odorant binding (Figure 5). As is shown in Supplementary Table 3, by using the KEGG database, the significant pathways were found to mainly contain immune response (hematopoietic cell lineage, antigen processing and presentation, complement and coagulation cascades, cytosolic DNA-sensing pathway, and Toll-like receptor signaling pathway); signaling molecules and interaction (ECM-receptor interaction, cytokine-cytokine receptor interaction, and neuroactive ligand-receptor interaction); signal transduction (TGF-beta signaling pathway and MAPK signaling pathway); cardiovascular diseases (dilated cardiomyopathy and hypertrophic cardiomyopathy); transport and catabolism (lysosome); cell communication (focal adhesion); and several pathways associated with the biosynthesis of other secondary metabolites, parasitic infectious diseases, and nervous system and nucleotide metabolism.

## Discussion

We investigated in the present study the effects of long-term consumption of RPS on the mucosa transcriptome, digesta- and mucosa-associated microbiota composition in the proximal colon of pigs. We found that compared with the control diet, the RPS diet changed the colonic expression profile of the host gene involved in immune response pathways; decreased the number of total bacteria and the relative abundance of *Clostridium, Treponema, Oscillospira, Phascolarctobacterium*, RC9 gut group, and S24-7-related OTUs; and increased the relative abundance of *Turicibacter, Blautia, Ruminococcus, Coprococcus, Marvinbryantia*, and *Ruminococcus bromii*-related OTUs.

Most studies today focus on the microbiota in the digesta of the hindgut or feces, whereas research on the effect of RS on mucosa-associated microbiota is limited. Our previous study showed that the substitution of about half of CS with RPS in pig diets increased the amount of RS in the diet, and the amount of starch in the cecum and colon of pigs (Fang et al., 2014). The present study is the first to our knowledge to use a high-throughput technique to investigate the RS effect on colonic mucosa-associated microbiota in pigs. In the present study, consumption of an RPS diet didn't affect the abundances of phyla, both in the digestive and mucosal microbiota. Martínez et al. (2010) reported that type RS4 but not RS2 significantly induced Bacteroidets and Actinobacteria while decreasing Firmicutes at the phylum level. Young et al. (2012), however, found blooms of Bacteroidets and Actinobacteria in colonic digesta when feeding type 2 RS to rats. The inconsistent results from the different studies may be due to different type of RS being used in different animal models.

The abundance of genera *Oscillospira, Parabacteroides, Phascolarctobacterium*, S24-7, *Alloprevotalla, Anaerotruncus*, RF16, *Bacteroides, Spirochaeta, Mucispirillum, Oscillibacter*, dgA-11 gut group, *Sutterella*, and *Mogibacterium* in colonic mucosa was significantly higher than that in digesta, while genera *Turicibacter, Anaerotruncu*, and Peptostreptococcaceae incertae_sediss showed the inverse tendency. In addition, we found that the richness estimators (ACE and Chao) of colonic mucosa-associated microbiota were higher than colonic lumen, which is in agreement with the results of a recent study where the mucosa-associated ileal microbiota harbored greater bacterial diversity than the lumen but similar membership to the mucosa of the large intestine in pigs (Looft et al., 2014). Meanwhile, we found that the RPS had the similar modulation effects on either relative abundance or diversity of mucosa-associated microbiota to the lumen in the colon of pigs, which suggests that the RS can affect the pigs through the interaction between mucosa-associated microbiota and the host cell, not just its fermentation products.

The genus *Ruminococcus*, which belong to clostridial cluster IV are numerically abundant in the large intestine of humans and pigs, typically accounting for 10–40% of total bacterial 16S rRNA sequences, but are under-represented by cultured isolates (Lay et al., 2005). There are strong indications, however, that this genus plays a primary role in the degradation of particulate substrates such as fiber and RS (Flint et al., 2008; Chassard et al., 2012). Recently, some *Ruminococcus* species were found to play a primary role in the degradation of dietary RS. Ze et al. (2012) found that *Ruminococcus bromii* is a keystone species for the degradation of RS in the human colon. Recent dietary intervention studies have reported increased populations of *R. bromii* and other gram-positive bacteria in fecal or colonic samples during consumption of diets high in RS (Abell et al., 2008; Martínez et al., 2010; Walker et al., 2011; Haenen et al., 2013b). Similarly, the present study also showed that the abundance of this species increased significantly in both colonic digesta and mucosa of pigs fed an RPS diet compared to the CS diet. In addition, we also found that the long-term intake of RPS increased the abundance of genus *Coprococcus* in the colon of pigs. Strains related to *Coprococcus* that belonged to the *Clostridium coccoides* cluster were also listed in major butyrate-producing bacteria isolated from the human colon in a previous review (Louis and Flint, 2009). The increase in the abundance of butyrate-producing bacteria is in agreement with the fact that an RPS diet significantly increased the concentrations of butyrate and total SCFA, as was shown in our preliminary study (Fang et al., 2014).

**Figure 6:**
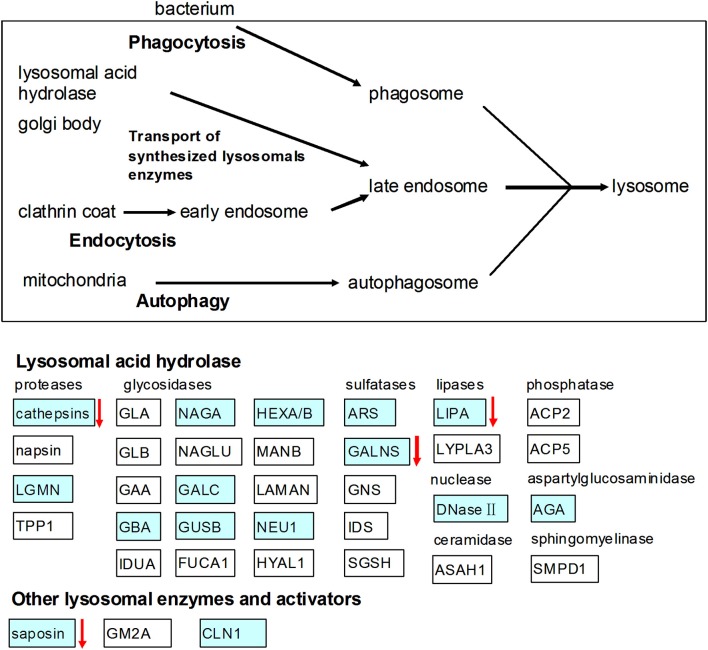
**Effects of high RS diet on lysosome pathway**. Gene symbols in blue indicate genes that are detected by microarray, genes indicated with arrows were significantly down-regulated.

In the present study, we found that RPS diet decreased the pH value in the colon contents of pigs, which may be due to the increase of SCFAs produced by microbial fermentation of starch (Fang et al., 2014). In addition, the lactate concentration in colon of the RPS group was also higher than the CS group (data no shown). The array data showed that gene expression of TLR-7 in the colon was down-regulated by the consumption of RPS. TLR7 recognizes single-stranded RNA in endosomes, which is a common feature of viral genomes that are internalized by macrophages and dendritic cells (Hipp et al., 2013). The decline in the pH value may result in the decrease of bacteria or virus populations in the colon of pigs. This was confirmed by the results of real-time PCR quantification of the complete 16S rRNA gene copies of total bacteria and the rarefaction curves of the 16S rRNA gene sequences. The array data in the present study also showed that RPS significantly suppressed genes involved in lysosome (Figure 6). Lysosomes are cellular organelles that contain acid hydrolase enzymes that break down waste materials and cellular debris. One of the major functions of lysosomes is the digestion of material taken up from outside the cell by endocytosis including excess or worn-out organelles, food particles, and engulfed viruses or bacteria. Similarly, Haenen et al. (2013b) found that short-term (2 weeks) intake of RS could also decrease the relative abundance of potential pathogens in the bacterial community, and could suppressed genes involved in both the innate and the adaptive immune response.

Recent studies have shown that the microbial metabolites, SCFA, can regulate colonic regulatory T cell homeostasis and can control gut inflammatory responses (Arpaia and Rudensky, 2014). Butyrate can suppress pro-inflammatory cytokine production by intestinal macrophages (Arpaia et al., 2013; Chang et al., 2014). The suppression role in immune response of a high RS diet may be due to the increase in SCFA. The array data in the current study showed, however, that long-term intake of RPS significantly increased pro-inflammatory cytokine IL-1β gene expression, which is not in line with the observation that the RPS increased the total SCFA and butyrate production in the colon in our previous study (Fang et al., 2014). The possible reason is that the significant decrease of pH value in the colon caused by long-term intake of PRS may result in the death of bacteria, and the accumulation of lipopolysaccharides (LPS). Similarly, the LPS have received increasing attention in rumen acidosis caused by a high concentration (starch) diet because of their role in the inflammatory response of the body (Tao et al., 2014). Although their benefit roles such as improving colonic mucosal integrity and reducing gut apoptosis are well known in humans and animals consuming a high RS diet (Nofrarías et al., 2007), the results of this study suggest that long-term intake of RPS may result in a negative effect on the health of pigs because of the low pH in the hindgut. Further studies are needed, however, to know whether this negative role will happen when using other type of RS diet or in humans and other animals in the long-term.

In conclusion, we have shown that long-term consumption of a diet high in RS modulated digesta and mucosa-associated microbial composition, and gene expression in the colon of pigs. While our findings suggest that a high RS diet may result in both positive and negative effects on gut health, which should not be ignored in further studies, additional investigation is required to further elucidate the underlying mechanisms of balancing the roles of RS on animal health.

## Author contributions

Conceived and designed the experiments: YS, WZ. Performed the experiments: YS, YS, LF, LZ. Analyzed the data: YS, LF, YS. Wrote the paper: YS, YS.

### Conflict of interest statement

The authors declare that the research was conducted in the absence of any commercial or financial relationships that could be construed as a potential conflict of interest.
