# Supplementary material for: Responses in colonic microbial community and gene expression of pigs to a long-term high resistant starch diet
Source: Front Microbiol. 2015 Aug 25;6:877. doi: 10.3389/fmicb.2015.00877 (PMC4548152; doi:10.3389/fmicb.2015.00877)
Supplement: Supplementary file 1 [file Table1.DOC]

***Supplementary Material***

**Responses in colonic microbial community and gene expression of pigs to a long-term high resistant starch diet**

Yue Sun, Liping Zhou, Lingdong Fang, Yong Su*, Weiyun Zhu

* **Correspondence:** Corresponding Author: yong.su@njau.edu.cn

**Supplementary Tables**

**Supplementary Table 1.** Relative abundances of microbial OTUs (percentage) that were significantly affected by the dietary treatment or the niche compartment in the colon of pigs^1^.

| OUT names | Colonic digesta | | Colonic mucosa | | SEM^2^ | *P* value | | Q value | | Annotation^3^ |
| --- | --- | --- | --- | --- | --- | --- | --- | --- | --- | --- |
|  | CS | RPS | CS | RPS |  | Niche compartment | Dietary | Niche compartment | Dietary |  |
| OTU496 | 13.23 | 5.449 | 11.06 | 5.014 | 6.336 | 0.565 | 0.006 | 0.840 | 0.037 | g:*Clostridium* |
| OTU164 | 6.740 | 6.514 | 3.523 | 3.648 | 3.019 | 0.014 | 0.959 | 0.099 | 0.959 | s:*Clostridium glycolicum* |
| OTU554 | 4.061 | 9.209 | 2.372 | 3.469 | 3.572 | 0.004 | 0.009 | 0.065 | 0.039 | g:*Turicibacter* |
| OTU129 | 2.882 | 6.491 | 1.878 | 3.047 | 2.336 | 0.006 | 0.002 | 0.070 | 0.022 | f:*Peptostreptococcaceae* |
| OTU263 | 0.674 | 3.174 | 0.705 | 2.249 | 2.299 | 0.639 | 0.033 | 0.871 | 0.090 | f:*Ruminococcaceae* |
| OTU74 | 0.106 | 1.584 | 0.084 | 3.097 | 2.267 | 0.415 | 0.017 | 0.718 | 0.050 | g:*Ruminococcus* |
| OTU56 | 0.001 | 2.354 | 0.000 | 1.930 | 2.379 | 0.829 | 0.030 | 0.911 | 0.086 | g:*Blautia* |
| OTU951 | 0.023 | 1.712 | 0.063 | 2.102 | 2.172 | 0.812 | 0.042 | 0.900 | 0.099 | f:*Lachnospiraceae* |
| OTU13 | 1.851 | 0.062 | 0.933 | 0.448 | 1.187 | 0.513 | 0.017 | 0.823 | 0.055 | g:*Treponema* |
| OTU435 | 0.138 | 1.207 | 0.234 | 1.050 | 0.963 | 0.947 | 0.017 | 0.972 | 0.048 | s:*Ruminococcus bromii* |
| OTU247 | 0.000 | 1.285 | 0.002 | 1.180 | 1.310 | 0.923 | 0.023 | 0.972 | 0.071 | g:*Ruminococcus* |
| OTU258 | 1.178 | 0.000 | 0.774 | 0.002 | 0.914 | 0.534 | 0.008 | 0.838 | 0.037 | g:*Treponema* |
| OTU194 | 0.156 | 0.983 | 0.176 | 0.657 | 0.480 | 0.326 | < 0.001 | 0.623 | 0.004 | f:*Christensenellaceae* |
| OTU378 | 0.100 | 0.002 | 1.269 | 0.018 | 0.672 | 0.008 | 0.006 | 0.076 | 0.037 | f:S24-7 |
| OTU395 | 0.637 | 0.026 | 0.648 | 0.026 | 0.379 | 0.953 | < 0.001 | 0.972 | 0.000 | f:*Ruminococcaceae* |
| OTU506 | 0.567 | 0.188 | 0.307 | 0.155 | 0.256 | 0.103 | 0.006 | 0.350 | 0.037 | g:*Clostridium* |
| OTU637 | 0.199 | 0.559 | 0.139 | 0.237 | 0.212 | 0.008 | 0.001 | 0.076 | 0.018 | f:*Christensenellaceae* |
| OTU209 | 0.094 | 0.030 | 0.741 | 0.140 | 0.323 | < 0.001 | 0.001 | 0.004 | 0.013 | g:*Phascolarctobacterium* |
| OTU136 | 0.407 | 0.039 | 0.362 | 0.157 | 0.268 | 0.739 | 0.007 | 0.881 | 0.037 | f:S24-7 |
| OTU827 | 0.551 | 0.001 | 0.324 | 0.000 | 0.373 | 0.362 | 0.002 | 0.664 | 0.023 | f:*Christensenellaceae* |
| OTU182 | 0.186 | 0.032 | 0.491 | 0.161 | 0.259 | 0.018 | 0.012 | 0.116 | 0.046 | g:RC9 gut group |
| OTU23 | 0.533 | 0.036 | 0.208 | 0.077 | 0.359 | 0.284 | 0.028 | 0.594 | 0.082 | g:p1088 a5 gut group |
| OTU842 | 0.122 | 0.017 | 0.572 | 0.150 | 0.239 | < 0.001 | < 0.001 | 0.002 | 0.004 | g:*Parabacteroides* |
| OTU238 | 0.026 | 0.027 | 0.265 | 0.558 | 0.339 | 0.005 | 0.254 | 0.070 | 0.329 | f:*Prevotellaceae* |
| OTU530 | 0.296 | 0.002 | 0.356 | 0.019 | 0.277 | 0.691 | 0.004 | 0.871 | 0.030 | g:*Treponema* |
| OTU240 | 0.008 | 0.463 | 0.008 | 0.171 | 0.363 | 0.328 | 0.034 | 0.623 | 0.090 | f:*Lachnospiraceae* |
| OTU323 | 0.034 | 0.005 | 0.496 | 0.086 | 0.276 | 0.006 | 0.031 | 0.070 | 0.087 | f:*Prevotellaceae* |
| OTU185 | 0.046 | 0.007 | 0.384 | 0.135 | 0.179 | < 0.001 | 0.008 | 0.003 | 0.037 | g:*Oscillospira* |
| OTU86 | 0.012 | 0.002 | 0.492 | 0.034 | 0.253 | 0.002 | 0.009 | 0.048 | 0.039 | g:*Bacteroides* |
| OTU426 | 0.032 | 0.208 | 0.064 | 0.268 | 0.191 | 0.536 | 0.015 | 0.838 | 0.054 | f:*Lachnospiraceae* |
| OTU284 | 0.321 | 0.014 | 0.166 | 0.029 | 0.186 | 0.245 | 0.002 | 0.554 | 0.019 | g:RC9 gut group |
| OTU931 | 0.040 | 0.310 | 0.031 | 0.161 | 0.151 | 0.102 | < 0.001 | 0.350 | 0.004 | f:*Peptostreptococcaceae* |
| OTU911 | 0.008 | 0.278 | 0.007 | 0.257 | 0.304 | 0.930 | 0.042 | 0.972 | 0.099 | g:*Ruminococcus* |
| OTU636 | 0.077 | 0.025 | 0.298 | 0.118 | 0.156 | 0.006 | 0.041 | 0.070 | 0.099 | g:*Parabacteroides* |
| OTU786 | 0.184 | 0.000 | 0.310 | 0.000 | 0.173 | 0.197 | < 0.001 | 0.478 | 0.004 | g:RC9 gut group |
| OTU205 | 0.059 | 0.246 | 0.040 | 0.087 | 0.144 | 0.124 | 0.034 | 0.378 | 0.090 | f:*Erysipelotrichaceae* |
| OTU433 | 0.182 | 0.002 | 0.220 | 0.006 | 0.198 | 0.771 | 0.016 | 0.897 | 0.055 | f:*Ruminococcaceae* |
| OTU883 | 0.035 | 0.184 | 0.060 | 0.141 | 0.100 | 0.834 | 0.003 | 0.911 | 0.027 | f:*Lachnospiraceae* |
| OTU233 | 0.333 | 0.004 | 0.047 | 0.005 | 0.192 | 0.033 | 0.008 | 0.164 | 0.037 | f:*Ruminococcaceae* |
| OTU568 | 0.039 | 0.002 | 0.306 | 0.036 | 0.166 | 0.008 | 0.012 | 0.076 | 0.046 | g:RC9 gut group |
| OTU17 | 0.029 | 0.005 | 0.246 | 0.106 | 0.136 | 0.001 | 0.079 | 0.030 | 0.144 | g:*Spirochaeta* |
| OTU514 | 0.124 | 0.029 | 0.160 | 0.059 | 0.085 | 0.267 | 0.003 | 0.588 | 0.027 | f:*Lachnospiraceae* |
| OTU168 | 0.077 | 0.001 | 0.272 | 0.006 | 0.135 | 0.013 | < 0.001 | 0.097 | 0.006 | g:*Prevotella* |
| OTU500 | 0.148 | 0.032 | 0.141 | 0.019 | 0.091 | 0.741 | 0.001 | 0.881 | 0.013 | f:*Ruminococcaceae* |
| OTU401 | 0.034 | 0.093 | 0.018 | 0.225 | 0.115 | 0.186 | 0.004 | 0.461 | 0.028 | g:*Blautia* |
| OTU609 | 0.037 | 0.147 | 0.054 | 0.100 | 0.092 | 0.713 | 0.037 | 0.871 | 0.096 | f:*Erysipelotrichaceae* |
| OTU589 | 0.007 | 0.008 | 0.168 | 0.148 | 0.092 | < 0.001 | 0.699 | 0.000 | 0.748 | f:*Prevotellaceae* |
| OTU99 | 0.010 | 0.007 | 0.241 | 0.055 | 0.129 | 0.002 | 0.042 | 0.048 | 0.099 | g:*Bacteroides* |
| OTU473 | 0.020 | 0.001 | 0.244 | 0.022 | 0.139 | 0.013 | 0.022 | 0.097 | 0.068 | g:*Parabacteroides* |
| OTU341 | 0.140 | 0.000 | 0.139 | 0.000 | 0.129 | 0.993 | 0.008 | 0.996 | 0.037 | g:RC9 gut group |
| OTU173 | 0.221 | 0.000 | 0.057 | 0.000 | 0.138 | 0.090 | 0.008 | 0.335 | 0.037 | f:*Ruminococcaceae* |
| OTU65 | 0.040 | 0.013 | 0.145 | 0.077 | 0.071 | 0.001 | 0.042 | 0.024 | 0.099 | g:*Oscillospira* |
| OTU11 | 0.001 | 0.067 | 0.006 | 0.218 | 0.126 | 0.105 | 0.006 | 0.350 | 0.036 | g:*Coprococcus* |
| OTU59 | 0.052 | 0.094 | 0.027 | 0.093 | 0.063 | 0.589 | 0.042 | 0.840 | 0.099 | f:*Lachnospiraceae* |
| OTU159 | 0.052 | 0.002 | 0.185 | 0.012 | 0.103 | 0.036 | 0.004 | 0.169 | 0.028 | g:RC9 gut group |
| OTU965 | 0.168 | 0.000 | 0.079 | 0.000 | 0.124 | 0.321 | 0.013 | 0.623 | 0.047 | f:*Christensenellaceae* |
| OTU962 | 0.005 | 0.105 | 0.015 | 0.142 | 0.127 | 0.649 | 0.034 | 0.871 | 0.090 | f:*Erysipelotrichaceae* |
| OTU337 | 0.132 | 0.000 | 0.109 | 0.000 | 0.115 | 0.778 | 0.010 | 0.897 | 0.042 | g:*Ruminococcus* |
| OTU651 | 0.185 | 0.000 | 0.047 | 0.000 | 0.099 | 0.031 | 0.001 | 0.162 | 0.014 | f:*Lachnospiraceae* |
| OTU160 | 0.026 | 0.107 | 0.017 | 0.099 | 0.058 | 0.620 | < 0.001 | 0.853 | 0.004 | g:*Marvinbryantia* |
| OTU397 | 0.086 | 0.011 | 0.109 | 0.027 | 0.066 | 0.388 | 0.002 | 0.703 | 0.020 | f:*Ruminococcaceae* |
| OTU58 | 0.005 | 0.125 | 0.005 | 0.103 | 0.072 | 0.595 | < 0.001 | 0.840 | 0.002 | g:*Coprococcus* |
| OTU250 | 0.003 | 0.000 | 0.126 | 0.105 | 0.109 | 0.010 | 0.779 | 0.084 | 0.818 | g:*Mucispirillum* |
| OTU158 | 0.113 | 0.008 | 0.076 | 0.021 | 0.071 | 0.612 | 0.004 | 0.849 | 0.028 | f:*Ruminococcaceae* |
| OTU55 | 0.150 | 0.012 | 0.037 | 0.017 | 0.092 | 0.111 | 0.025 | 0.357 | 0.074 | f:*Ruminococcaceae* |
| OTU131 | 0.003 | 0.006 | 0.104 | 0.112 | 0.098 | 0.010 | 0.884 | 0.084 | 0.907 | f:*Prevotellaceae* |
| OTU699 | 0.005 | 0.000 | 0.176 | 0.025 | 0.102 | 0.008 | 0.043 | 0.076 | 0.099 | g:dgA-11 gut group |
| OTU366 | 0.023 | 0.089 | 0.035 | 0.065 | 0.047 | 0.783 | 0.012 | 0.897 | 0.046 | f:*Lachnospiraceae* |
| OTU817 | 0.108 | 0.010 | 0.074 | 0.009 | 0.083 | 0.575 | 0.015 | 0.840 | 0.054 | g:*Blautia* |
| OTU970 | 0.032 | 0.000 | 0.162 | 0.002 | 0.085 | 0.015 | 0.002 | 0.099 | 0.020 | f:*Prevotellaceae* |
| OTU937 | 0.010 | 0.000 | 0.184 | 0.000 | 0.106 | 0.019 | 0.017 | 0.116 | 0.055 | g:*Bacteroides* |
| OTU748 | 0.049 | 0.003 | 0.109 | 0.037 | 0.062 | 0.033 | 0.011 | 0.164 | 0.046 | g:*Anaerotruncus* |
| OTU942 | 0.088 | 0.032 | 0.050 | 0.027 | 0.041 | 0.155 | 0.014 | 0.418 | 0.052 | f:Family XIII |

## ^1^OTUs with relative abundances higher than 0.05% within total bacteria were sorted and showed in the table.

## ^2^SEM, standard error of means, n=5 or 6.

^3^The consensus sequence of each OTU was annotated to the closest lineage using MOTHUR program against the SILVA 16S rRNA reference database. s: = species; g: = genus; f: = family; o: = order**.**
