# Supplementary material for: Responses in colonic microbial community and gene expression of pigs to a long-term high resistant starch diet
Source: Front Microbiol. 2015 Aug 25;6:877. doi: 10.3389/fmicb.2015.00877 (PMC4548152; doi:10.3389/fmicb.2015.00877)
Supplement: Supplementary file 2 [file Table2.DOC]

***Supplementary Material***

**Responses in colonic microbial community and gene expression of pigs to a long-term high resistant starch diet**

Yue Sun, Liping Zhou, Lingdong Fang, Yong Su*, Weiyun Zhu

* **Correspondence:** Corresponding Author: yong.su@njau.edu.cn

**Supplementary Table 2.** Differently expressed genes (P < 0.05, fold change > 2) in the colonic mucosa of pigs between two dietary groups (RPS diet vs CS diet).

| Probe name | *P* value | Fold change | Gene symbol | Gene name |
| --- | --- | --- | --- | --- |
| A_72_P165196 | 0.017 | 4.05 | F7 | coagulation factor VII (serum prothrombin conversion accelerator) |
| A_72_P733318 | 0.003 | 3.10 | HFE2 | hemochromatosis type 2 (juvenile) |
| A_72_P012811 | 0.004 | 3.06 | LOC100515970 | oxysterol-binding protein 2-like |
| A_72_P491792 | 0.020 | 2.77 | LOC100522289 | gamma-aminobutyric acid receptor subunit rho-2-like |
| A_72_P441509 | 0.009 | 2.76 | PMAP-23 | antibacterial protein |
| A_72_P146626 | 0.008 | 2.67 | CPEB1 | cytoplasmic polyadenylation element binding protein 1 |
| A_72_P440216 | 0.001 | 2.66 | CASS4 | Cas scaffolding protein family member 4 |
| A_72_P407323 | 0.001 | 2.58 | C1QB | complement component 1, q subcomponent, B chain |
| A_72_P293829 | 0.012 | 2.54 | COBLL1 | COBL-like 1 |
| A_72_P180436 | 0.041 | 2.53 | F2R | coagulation factor II (thrombin) receptor |
| A_72_P478879 | 0.039 | 2.53 | LOC100623005 | uncharacterized protein C14orf43-like |
| A_72_P555980 | 0.005 | 2.52 | LOC100157115 | Rhabdoid tumor deletion region gene 1 |
| A_72_P444051 | 0.043 | 2.40 | F2R | coagulation factor II (thrombin) receptor |
| A_72_P104536 | 0.017 | 2.36 | APLP2 | amyloid beta (A4) precursor-like protein 2 |
| A_72_P477770 | 0.031 | 2.36 | LOC100156508 | secretory carrier-associated membrane protein 5-like |
| A_72_P124881 | 0.045 | 2.32 | LOC100524932 | protein kinase C epsilon type-like |
| A_72_P263242 | 0.022 | 2.26 | NTRK1 | neurotrophic tyrosine kinase, receptor, type 1 |
| A_72_P463511 | 0.001 | 2.20 | IL-1beta | interleukin-1 beta |
| A_72_P494059 | 0.011 | 2.17 | SIX1 | SIX homeobox 1 |
| A_72_P177816 | 0.009 | 2.17 | MMP7 | matrix metallopeptidase 7 (matrilysin, uterine) |
| A_72_P224337 | 0.000 | 2.15 | LOC654291 | SCL2A4RG-like |
| A_72_P488589 | 0.008 | 2.14 | LOC100152038 | oncostatin-M-like |
| A_72_P356028 | 0.000 | 2.12 | CLDN15 | claudin 15 |
| A_72_P688749 | 0.013 | 2.09 | NPG4 | protegrin 4 |
| A_72_P165820 | 0.049 | 2.08 | AVPR2 | arginine vasopressin receptor 2 |
| A_72_P701260 | 0.016 | 2.07 | ZNF268 | zinc finger protein 268 |
| A_72_P002511 | 0.018 | 2.02 | AMH | anti-Mullerian hormone |
| A_72_P035871 | 0.012 | 2.00 | NPG4 | protegrin 4 |
| A_72_P209407 | 0.013 | 0.50 | SERPING1 | serpin peptidase inhibitor, clade G (C1 inhibitor), member 1 |
| A_72_P178086 | 0.025 | 0.49 | CTSD | cathepsin D |
| A_72_P772656 | 0.009 | 0.49 | CTSD | cathepsin D |
| A_72_P638165 | 0.041 | 0.48 | SPARC | secreted protein, acidic, cysteine-rich (osteonectin) |
| A_72_P640386 | 0.022 | 0.48 | FST | follistatin |
| A_72_P528376 | 0.010 | 0.47 | LOC100152849 | EMI domain-containing protein 1-like |
| A_72_P489510 | 0.023 | 0.47 | PLAC9 | placenta-specific 9 |
| A_72_P203952 | 0.008 | 0.46 | BGN | biglycan |
| A_72_P669264 | 0.010 | 0.46 | AXL | AXL receptor tyrosine kinase |
| A_72_P621617 | 0.043 | 0.46 | TGFB3 | transforming growth factor, beta 3 |
| A_72_P466686 | 0.000 | 0.45 | PLS3 | plastin 3 |
| A_72_P639642 | 0.035 | 0.45 | SPARC | secreted protein, acidic, cysteine-rich (osteonectin) |
| A_72_P516034 | 0.017 | 0.45 | FLNA | filamin A, alpha |
| A_72_P058151 | 0.038 | 0.44 | SLC45A2 | solute carrier family 45, member 2 |
| A_72_P351123 | 0.030 | 0.44 | FGL2 | fibrinogen-like 2 |
| A_72_P209987 | 0.042 | 0.44 | ADORA3 | adenosine A3 receptor |
| A_72_P704216 | 0.008 | 0.43 | LOC100156093 | tumor suppressor candidate 3-like |
| A_72_P442926 | 0.036 | 0.43 | C1QB | complement component 1, q subcomponent, B chain |
| A_72_P403253 | 0.040 | 0.43 | C1QC | complement component 1, q subcomponent, C chain |
| A_72_P442355 | 0.010 | 0.41 | LOC396903 | myosin |
| A_72_P440951 | 0.050 | 0.39 | MMP9 | matrix metallopeptidase 9 (gelatinase B, 92kDa gelatinase, 92kDa type IV collagenase) |
| A_72_P592214 | 0.017 | 0.38 | ADORA3 | adenosine A3 receptor |
| A_72_P441424 | 0.035 | 0.35 | GAL | galanin/GMAP prepropeptide |
| A_72_P561029 | 0.038 | 0.33 | FHL1C | four and a half LIM domains 1 protein, isoform C |
| A_72_P615191 | 0.019 | 0.31 | FHL1C | four and a half LIM domains 1 protein, isoform C |
| A_72_P165031 | 0.036 | 0.28 | TLR7 | toll-like receptor 7 |
| A_72_P302634 | 0.021 | 0.24 | DUOX1 | dual oxidase 1 |

**Supplementary Table 3.** The KEGG Pathways enriched with differentially expressed genes induced by dietary treatment (RPS diet vs CS diet).

| Pathway database | Pathway name | Gene | Change | [Hits](javascript:void(0);) | [Total](javascript:void(0);) | [Percent](javascript:void(0);) | [Enrichment test *P* value](javascript:void(0);) | [Q value](javascript:void(0);) |
| --- | --- | --- | --- | --- | --- | --- | --- | --- |
| Immune system | Hematopoietic cell lineage | CD4 | Down | 3 | 63 | 4.76% | 0.0098 | 0.0048 |
|  |  | IL-1B | Up |  |  |  |  |  |
|  |  | ITGB3 | Down |  |  |  |  |  |
|  | Antigen processing and presentation | CD4 | Down | 3 | 64 | 4.69% | 0.0102 | 0.0048 |
|  |  | CTSB | Down |  |  |  |  |  |
|  |  | CTSB/L/S | Down |  |  |  |  |  |
|  | Complement and coagulation cascades | C1S | Down | 3 | 65 | 4.62% | 0.0106 | 0.0048 |
|  |  | F7 | Up |  |  |  |  |  |
|  |  | SERPING1 | Down |  |  |  |  |  |
|  | Cytosolic DNA-sensing pathway | IL-1B | Up | 2 | 48 | 4.17% | 0.0439 | 0.0115 |
|  |  | PolⅢ | Up |  |  |  |  |  |
|  | Toll-like receptor signaling pathway | IL-1B | Up | 3 | 74 | 4.05% | 0.0149 | 0.0062 |
|  |  | TLR6 | Up |  |  |  |  |  |
|  |  | TLR7 | Down |  |  |  |  |  |
| Signaling molecules and interaction | ECM-receptor interaction | COL5A2 | Down | 3 | 43 | 6.98% | 0.0036 | 0.0036 |
|  |  | COL6A3 | Down |  |  |  |  |  |
|  |  | ITGB3 | Down |  |  |  |  |  |
|  | Cytokine-cytokine receptor interaction | AMH | Up | 4 | 142 | 2.82% | 0.0167 | 0.0064 |
|  |  | IL-1B | Up |  |  |  |  |  |
|  |  | TGFB3 | Down |  |  |  |  |  |
|  |  | OSM | Up |  |  |  |  |  |
|  | Neuroactive ligand-receptor interaction | GAL | Down | 4 | 174 | 2.3% | 0.0316 | 0.0093 |
|  |  | CALCRL | Up |  |  |  |  |  |
|  |  | AVPR2 | Up |  |  |  |  |  |
|  |  | ADORA3 | Down |  |  |  |  |  |
| Signal transduction | TGF-beta signaling pathway | FST | Down | 3 | 55 | 5.45% | 0.0069 | 0.0048 |
|  |  | AMH | Up |  |  |  |  |  |
|  |  | TGFB3 | Down |  |  |  |  |  |
|  | MAPK signaling pathway | IL-1B | Up | 3 | 100 | 3.0% | 0.0317 | 0.0093 |
|  |  | TGFB3 | Down |  |  |  |  |  |
|  |  | NTRK1 | Up |  |  |  |  |  |
| Cardiovascular diseases | Dilated cardiomyopathy | ITGB3 | Down | 3 | 40 | 7.5% | 0.0030 | 0.0036 |
|  |  | TGFB3 | Down |  |  |  |  |  |
|  |  | TPM2 | Down |  |  |  |  |  |
|  | Hypertrophic cardiomyopathy | ITGB3 | Down | 3 | 43 | 6.98% | 0.0036 | 0.0036 |
|  |  | TGFB3 | Down |  |  |  |  |  |
|  |  | TPM2 | Down |  |  |  |  |  |
| Transport and catabolism | Lysosome | LIPA | Down | 7 | 65 | 10.77% | 0.000 | 0.000 |
|  |  | GALNS | Down |  |  |  |  |  |
|  |  | CTSZ | Down |  |  |  |  |  |
|  |  | CTSB | Down |  |  |  |  |  |
|  |  | CTSD | Down |  |  |  |  |  |
|  |  | CH242-247L10.3 | Down |  |  |  |  |  |
|  |  | PSAP | Down |  |  |  |  |  |
| Cell communication | Focal adhesion | COL5A2 | Down | 4 | 79 | 5.06% | 0.0023 | 0.0036 |
|  |  | COL6A3 | Down |  |  |  |  |  |
|  |  | ITGB3 | Down |  |  |  |  |  |
|  |  | ECM | UP |  |  |  |  |  |
| Biosynthesis of other secondary metabolites | Caffeine metabolism | CYP2A19 | Down | 1 | 5 | 20.0% | 0.0393 | 0.0109 |
| Cancers: Specific types | Chronic myeloid leukemia | TGFB3 | Down | 2 | 39 | 5.13% | 0.0305 | 0.0093 |
|  |  | SHC1 | Up |  |  |  |  |  |
| Infectious diseases: Parasitic | Leishmania infection | FCGR3B | Down | 3 | 63 | 4.76% | 0.0098 | 0.0048 |
|  |  | IL-1B | Up |  |  |  |  |  |
|  |  | TGFB3 | Down |  |  |  |  |  |
| Nervous system | Neurotrophin signaling pathway | NTRK1 | Up | 3 | 57 | 5.26% | 0.0075 | 0.0048 |
|  |  | SH2B3 | Up |  |  |  |  |  |
|  |  | SHC1 | Up |  |  |  |  |  |
| Nucleotide metabolism | Pyrimidine metabolism | ENTPD1 | Down | 2 | 30 | 6.67% | 0.0192 | 0.0069 |
|  |  | POLR3H | Up |  |  |  |  |  |
