# Supplementary material for: Responses in colonic microbial community and gene expression of pigs to a long-term high resistant starch diet
Source: Front Microbiol. 2015 Aug 25;6:877. doi: 10.3389/fmicb.2015.00877 (PMC4548152; doi:10.3389/fmicb.2015.00877)
Supplement: Supplementary file 3 [file Table3.DOC]

***Supplementary Material***

**Responses in colonic microbial community and gene expression of pigs to a long-term high resistant starch diet**

Yue Sun, Liping Zhou, Lingdong Fang, Yong Su*, Weiyun Zhu

* **Correspondence:** Corresponding Author: yong.su@njau.edu.cn

**Supplementary Table 3.** The KEGG Pathways enriched with differentially expressed genes induced by dietary treatment (RPS diet vs CS diet).

| Pathway database | Pathway name | Gene | Change | [Hits](javascript:void(0);) | [Total](javascript:void(0);) | [Percent](javascript:void(0);) | [Enrichment test *P* value](javascript:void(0);) | [Q value](javascript:void(0);) |
| --- | --- | --- | --- | --- | --- | --- | --- | --- |
| Immune system | Hematopoietic cell lineage | CD4 | Down | 3 | 63 | 4.76% | 0.0098 | 0.0048 |
|  |  | IL-1B | Up |  |  |  |  |  |
|  |  | ITGB3 | Down |  |  |  |  |  |
|  | Antigen processing and presentation | CD4 | Down | 3 | 64 | 4.69% | 0.0102 | 0.0048 |
|  |  | CTSB | Down |  |  |  |  |  |
|  |  | CTSB/L/S | Down |  |  |  |  |  |
|  | Complement and coagulation cascades | C1S | Down | 3 | 65 | 4.62% | 0.0106 | 0.0048 |
|  |  | F7 | Up |  |  |  |  |  |
|  |  | SERPING1 | Down |  |  |  |  |  |
|  | Cytosolic DNA-sensing pathway | IL-1B | Up | 2 | 48 | 4.17% | 0.0439 | 0.0115 |
|  |  | PolⅢ | Up |  |  |  |  |  |
|  | Toll-like receptor signaling pathway | IL-1B | Up | 3 | 74 | 4.05% | 0.0149 | 0.0062 |
|  |  | TLR6 | Up |  |  |  |  |  |
|  |  | TLR7 | Down |  |  |  |  |  |
| Signaling molecules and interaction | ECM-receptor interaction | COL5A2 | Down | 3 | 43 | 6.98% | 0.0036 | 0.0036 |
|  |  | COL6A3 | Down |  |  |  |  |  |
|  |  | ITGB3 | Down |  |  |  |  |  |
|  | Cytokine-cytokine receptor interaction | AMH | Up | 4 | 142 | 2.82% | 0.0167 | 0.0064 |
|  |  | IL-1B | Up |  |  |  |  |  |
|  |  | TGFB3 | Down |  |  |  |  |  |
|  |  | OSM | Up |  |  |  |  |  |
|  | Neuroactive ligand-receptor interaction | GAL | Down | 4 | 174 | 2.3% | 0.0316 | 0.0093 |
|  |  | CALCRL | Up |  |  |  |  |  |
|  |  | AVPR2 | Up |  |  |  |  |  |
|  |  | ADORA3 | Down |  |  |  |  |  |
| Signal transduction | TGF-beta signaling pathway | FST | Down | 3 | 55 | 5.45% | 0.0069 | 0.0048 |
|  |  | AMH | Up |  |  |  |  |  |
|  |  | TGFB3 | Down |  |  |  |  |  |
|  | MAPK signaling pathway | IL-1B | Up | 3 | 100 | 3.0% | 0.0317 | 0.0093 |
|  |  | TGFB3 | Down |  |  |  |  |  |
|  |  | NTRK1 | Up |  |  |  |  |  |
| Cardiovascular diseases | Dilated cardiomyopathy | ITGB3 | Down | 3 | 40 | 7.5% | 0.0030 | 0.0036 |
|  |  | TGFB3 | Down |  |  |  |  |  |
|  |  | TPM2 | Down |  |  |  |  |  |
|  | Hypertrophic cardiomyopathy | ITGB3 | Down | 3 | 43 | 6.98% | 0.0036 | 0.0036 |
|  |  | TGFB3 | Down |  |  |  |  |  |
|  |  | TPM2 | Down |  |  |  |  |  |
| Transport and catabolism | Lysosome | LIPA | Down | 7 | 65 | 10.77% | 0.000 | 0.000 |
|  |  | GALNS | Down |  |  |  |  |  |
|  |  | CTSZ | Down |  |  |  |  |  |
|  |  | CTSB | Down |  |  |  |  |  |
|  |  | CTSD | Down |  |  |  |  |  |
|  |  | CH242-247L10.3 | Down |  |  |  |  |  |
|  |  | PSAP | Down |  |  |  |  |  |
| Cell communication | Focal adhesion | COL5A2 | Down | 4 | 79 | 5.06% | 0.0023 | 0.0036 |
|  |  | COL6A3 | Down |  |  |  |  |  |
|  |  | ITGB3 | Down |  |  |  |  |  |
|  |  | ECM | UP |  |  |  |  |  |
| Biosynthesis of other secondary metabolites | Caffeine metabolism | CYP2A19 | Down | 1 | 5 | 20.0% | 0.0393 | 0.0109 |
| Cancers: Specific types | Chronic myeloid leukemia | TGFB3 | Down | 2 | 39 | 5.13% | 0.0305 | 0.0093 |
|  |  | SHC1 | Up |  |  |  |  |  |
| Infectious diseases: Parasitic | Leishmania infection | FCGR3B | Down | 3 | 63 | 4.76% | 0.0098 | 0.0048 |
|  |  | IL-1B | Up |  |  |  |  |  |
|  |  | TGFB3 | Down |  |  |  |  |  |
| Nervous system | Neurotrophin signaling pathway | NTRK1 | Up | 3 | 57 | 5.26% | 0.0075 | 0.0048 |
|  |  | SH2B3 | Up |  |  |  |  |  |
|  |  | SHC1 | Up |  |  |  |  |  |
| Nucleotide metabolism | Pyrimidine metabolism | ENTPD1 | Down | 2 | 30 | 6.67% | 0.0192 | 0.0069 |
|  |  | POLR3H | Up |  |  |  |  |  |
